# *Entamoeba bangladeshi* nov. sp., Bangladesh

**DOI:** 10.3201/eid1809.120122

**Published:** 2012-09

**Authors:** Tricia L. Royer, Carol Gilchrist, Mamun Kabir, Tuhinur Arju, Katherine S. Ralston, Rashidul Haque, C. Graham Clark, William A. Petri

**Affiliations:** University of Virginia School of Medicine, Charlottesville, Virginia, USA (T.L. Royer, C. Gilchrist, K.S. Ralston, W.A. Petri, Jr.);; International Centre for Diarrheal Disease Research, Dhaka, Bangladesh (M. Kabir, T. Arju, R. Haque);; and London School of Hygiene and Tropical Medicine, London, UK (C.G. Clark)

**Keywords:** Entamoeba, Entamoeba Bangladeshi, polymerase chain reaction, phylogeny, ribosomal RNA, Bangladesh, parasites

**To the Editor:** Diarrheal diseases have a major effect on global health, particularly the health of malnourished children (*1*). The enteric parasites *Entamoeba histolytica* and *E. moshkovskii* are potential causes of diarrheal disease in children (*2*). For the last 20 years, we have been studying *Entamoeba* infections in children from the urban slum of Mirpur in Dhaka, Bangladesh (*3*).

*E. histolytica* infections can be detected through fecal microscopy, culture, PCR, and antigen detection. Microscopy and culture have limited specificity because several species of *Entamoeba,* which vary in their pathogenic potential, have morphologically similar cysts and trophozoites (*4*). In 2010–2011, during analysis of feces positive for *Entamoeba* organisms by microscopy or culture but negative for *E. histolytica*, *E. dispar*, and *E. moshkovskii* by PCR, a new species was identified, which we have named *Entamoeba bangladeshi* nov. sp. in recognition of the support of the Bangladesh community for this research.

Feces from both diarrheal and surveillance specimens were collected from a cohort of children living in Mirpur (*3*). A total of 2,039 fecal samples were examined microscopically (0.9% saline smear) and/or by fecal culture for amebic trophozoites and cysts (*4*). One hundred forty-nine (7%) of the samples were positive by microscopy and/or culture for an *Entamoeba* parasite with both cysts and trophozoites that closely resembled those of *E. histolytica*, *E. moshkovskii*, and *E. dispar*.

DNA was extracted directly from fecal samples by using the QIAamp DNA Stool Mini Kit (QIAGEN, Hilden, Germany) according to the manufacturer’s instructions. DNA from positive fecal cultures was isolated by using the cetyl-trimethylammonium bromide extraction method (*5*). PCR was conducted to detect *E. histolytica*, *E. dispar*, and *E. moshkovskii*, all of which are morphologically indistinguishable by microscopy and are endemic to Bangladesh (Table) (*7*–*10*). An antigen detection test (TechLab Inc. Blacksburg, VA, USA) was also used to identify fecal samples positive for *E. histolytica*.

**Table:** Oligonucleotide primers used for screening and sequencing of *Entamoeba bangladeshi* nov. sp., Bangladesh*

Target organism	Primer name	Primer sequence, 5′ → 3′	Reference
Broad specificity *Entamoeba* sp.	Entagen-F	ACT TCA GGG GGA GTA TGG TCA C	(*7*)
	Entagen-R	CAA GAT GTC TAA GGG CAT CAC AG	(*7*)
*E. histolytica*	Eh-F	AAC AGT AAT AGT TTC TTT GGT TAG TAA AA	(*9*)
	Eh-R	CTT AGA ATG TCA TTT CTC AAT TCA T	(*9*)
	Eh-YYT Probe	**YYT-**ATT AGT ACA AAA TGG CCA ATT CAT TCA**-Dark Quencher**	(*9*)
*E. moshkovskii*	Em-1	CTC TTC ACG GGG AGT GCG	(*8*)
	Em-2	TCG TTA GTT TCA TTA CCT	(*8*)
	nEm-1	GAA TAA GGA TGG TAT GAC	(*8*)
	nEm-2	AAG TGG AGT TAA CCA CCT	(*8*)
*E. dispar*	E-1	TTT GTA TTA GTA CAA A	(*10*)
	E-2	GTA [A/G]TA TTG ATA TAC T	(*10*)
	Ed-1	AGT GGC CAA TTT ATG TAA GT	(*10*)
	Ed-2	TTT AGA AAC AAT GTT TCT TC	(*10*)

*Boldface indicates the probe fluorophore and quencher.

Fecal samples (129) and cultures derived from fecal material (20) were tested by PCR. Forty-four fecal samples were positive for *E. histolytica*, 42 for *E. dispar*, and 7 for *E. moshkovskii*. PCR results for 48 samples were negative for all 3 parasites (mixed infections account for the total being >129); 5 cultures also were negative for all 3 parasites.

ENTAGEN-F and ENTAGEN-R primers, which exhibit a broad specificity for the small subunit ribosomal RNA (SSU rRNA) gene sequences of *Entamoeba*, were used in PCR to amplify DNA fragments from 43 of the samples that were negative by PCR for the 3 *Entamoeba* species; amplification conditions were adapted from Stensvold et al. (*6*). The amplified DNA was separated by electrophoresis by using 2% agarose gel. Bands of the size predicted for the *Entamoeba* spp. SSU rRNA gene amplicon were detected in 15 samples (Technical Appendix Table). The PCR products were extracted by using the QIAquick Gel Extraction Kit (QIAGEN) and cloned by using the Zero Blunt TOPO Cloning Kit (Invitrogen, Carlsbad, CA, USA). The sequenced clones from 2 different isolates, 1 diarrheal and 1 surveillance specimen, were completely novel when compared with the SSU rRNA gene sequences from other organisms and did not match any previously sequenced *Entamoeba* species. These isolates represent a new species of *Entamoeba* (GenBank accession nos. JQ412861 and JQ412862), here named *E. bangladeshi* (Technical Appendix)

We examined the phylogenetic relationship between *E. bangladeshi* and other *Entamoeba* parasites by using maximum-likelihood analysis as implemented in MEGA 5 (Technical Appendix Figure, panel A). *E. bangladeshi*, although distinct, clearly grouped with the clade of *Entamoeba* infecting humans, including *E. histolytica*. *E. bangladeshi*, however, appeared more distantly related than the noninvasive *E. dispar*, but closer than *E. moshkovskii,* to *E. histolytica*.

To further characterize *E. bangladeshi,* we established it in xenic culture, and it displayed the ability to grow at 37°C and 25°C, a characteristic shared with *E. moshkovskii* and *E. ecuadoriensis* but that distinguishes it from *E. histolytica* and *E. dispar*. Cultured trophozoites were evaluated through light and transmission electron microscopy (Technical Appendix Figure, panel B). By light microscopy, we detected no apparent differences between *E. bangladeshi* and *E. histolytica*. The physical resemblance between *E. histolytica* and *E. bangladeshi* is notable because direct microscopic examination of fecal samples is still used as a diagnostic tool in areas to which these species are endemic to detect *E. histolytica* parasites.

Our findings add to the diversity of *Entamoeba* species found in humans. The incidence and effect of infection in infants by the newly recognized species *E. bangladeshi* await future epidemiologic studies.

Technical Appendix Sequencing outcomes for 13 samples that were negative by PCR for *Entamoeba histolytica*, *E. dispar*, and *E. moshkovskii* and taxonomic summary for *E. Bangladeshi*.
