# Supplementary material for: Entamoeba bangladeshi nov. sp., Bangladesh
Source: Emerg Infect Dis. 2012 Sep;18(9):1543–5. doi: 10.3201/eid1809.120122 (PMC3437720; doi:10.3201/eid1809.120122)
Supplement: Technical Appendix — Sequencing outcomes for 13 samples that were negative by PCR for Entamoeba histolytica, E. dispar, and E. moshkovskii and taxonomic summary for E. Bangladeshi. [file 12-0122-Techapp-s1.pdf]

Article DOI: http://dx.doi.org/10.3201/eid1809.120122

Entamoeba bangladeshi nov. sp., Bangladesh

Technical Appendix

Table. Sequencing outcomes for remaining 13 of 15 samples that were negative by PCR for *Entamoeba histolytica*, *E. dispar*, and *E. moshkovskii* and that were not the novel species *E. bangladeshi*

Specimen no.	Sequencing outcome
1	97% identity to *E. bangladeshi*, but insufficient sequence length led to ambiguous assignment, conservatively removed from further analysis
2	No priming upon sequencing
3	No priming upon sequencing
4	98% identity to *E. hartmanni,* 99% similar to specimen AM 385
5	Poor priming, produced sequence of insufficient length for analysis
6	98% identity to *E. hartmanni,* 99% similar to specimen AM 288
7	No priming upon sequencing
8	No priming upon sequencing
9	No priming upon sequencing
10	100% identity to *E. moshkovskii;* this was likely not picked up by species-specific qPCR secondary to degradation of the sample and loss of specific primer locus
11	No priming upon sequencing
12	No priming upon sequencing
13	No priming upon sequencing

Taxonomic Summary *Entamoeba bangladeshi*

Diagnosis. Microscopically indistinguishable from *Entamoeba histolytica* in cyst and trophozoite stages (*1*). In xenic culture, has the ability to grow at 37°C and room temperature, a characteristic shared with *E. moshkovskii* and *E. ecuadoriensis* but which distinguishes it from *E. histolytica* and *E. dispar*. Tests negative in *E. histolytica* ELISA and in species-specific PCRs. Currently only identifiable by its small subunit ribosomal RNA gene sequence.

Host/Type locality. Obtained from infant feces in Mirpur, Dhaka, Bangladesh, N 23°47′34,” E 90°21′38”

Etymology. Species name reflects the geographic origin of the specimen and recognizes the contribution of the Bangladeshi people to amebiasis research.

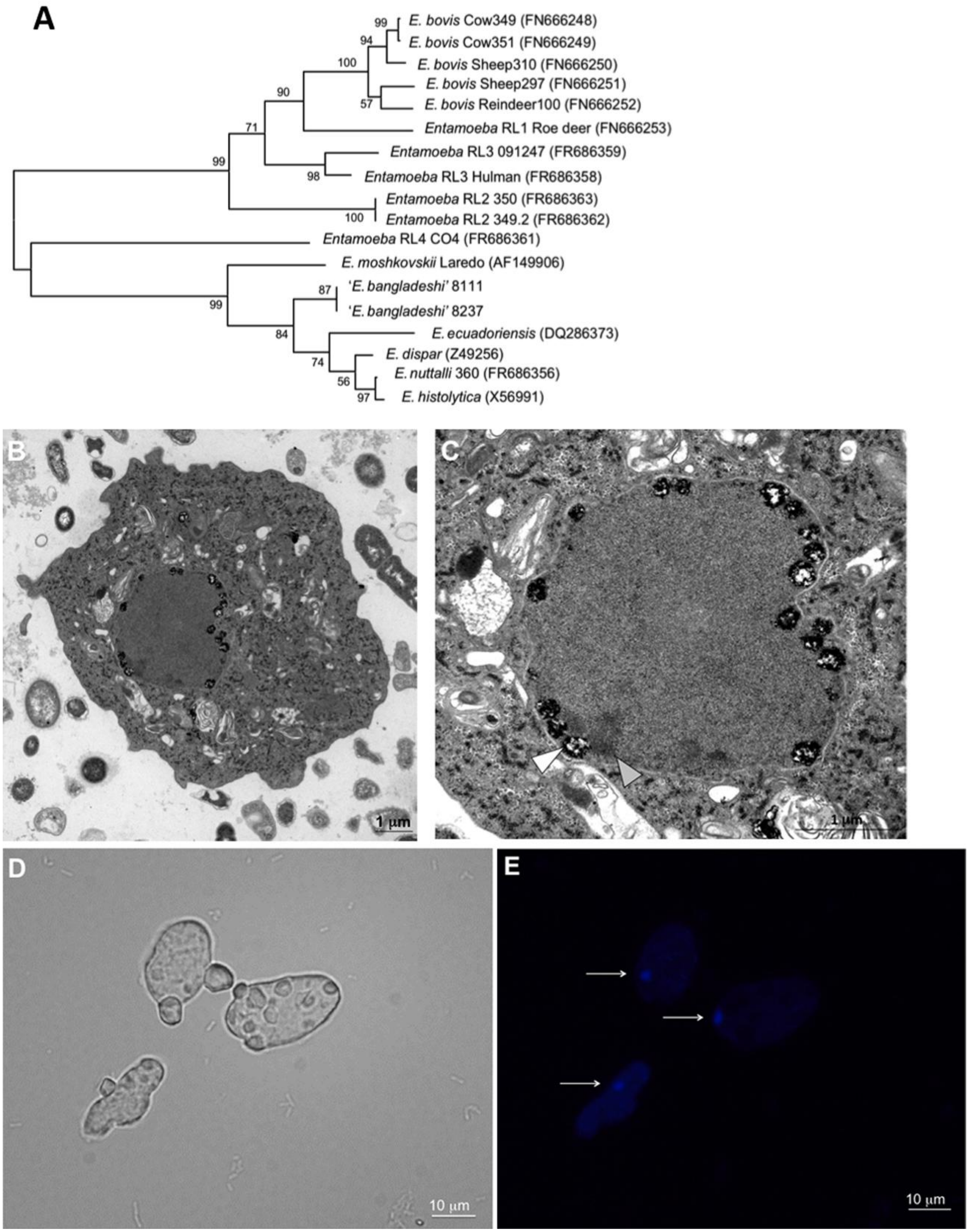

Figure. Phylogenetic relationships of *Entamoeba bangladeshi*. A) Phylogenetic analyses were performed by using maximum likelihood (MEGA 5 [*2*]) and a general time-reversible model of nucleotide substitution

with 4 γ-distributed categories of among-site rate variation and the proportion of invariant sites, selected by ModelTest (as implemented in MEGA 5). Statistical support was evaluated by using bootstrapping (1,000 replicates). Although unrooted, the tree is shown with the topology found by Stensvold et al. (*3*). The sequence alignment was derived from that used in Figure 2 of Stensvold et al. (*3*), with the remainder of the *E. bangladeshi* small subunit ribosomal gene sequence classified as missing data. Numbers in parentheses after species names are GenBank accession numbers. B) Transmission electron microscopy (TEM) demonstrating the ultrastructure of an *E. bangladeshi* trophozoite. Original magnification ×4,000. Scale bar = 1 μm. C) Higher magnification (×10,000) view of the nucleus of the *E. bangladeshi* trophozoite from panel B, illustrating the peripheral chromatin (gray arrowhead). *E. bangladeshi* and *E. histolytica* trophozoites have similar ultrastructure; however, the nucleus of *E. bangladeshi* presents more dark-staining structures (white arrowhead) than the typical *E. histolytica* nucleus. Scale bar = 1 μm. TEM samples were fixed in 2% paraformaldehyde and 2% glutaraldehyde in phosphate buffered saline and then postfixed with 1% osmium tetroxide plus 0.1% potassium ferrocyanide. Dehydration through an ethanol gradient was performed, followed by infiltration and embedment in epon. Sections were cut on a Leica Ultracut Ultramicrotome (Leica Microsystems Inc., Buffalo Grove, IL, USA), poststained with uranyl acetate and lead citrate, and imaged by using a JEOL 1230 transmission electron microscope (JEOL, Tokyo, Japan). D and E) Light microscopy analysis of *E. bangladeshi* trophozoites. Light microscopy samples were fixed with 4% paraformaldehyde and stained with DAPI (Invitrogen, Carlsbad, CA, USA) to visualize nuclei. Shown is a phase contrast image containing 3 amoebae (D) and the corresponding DAPI fluorescence (E). Nuclei are indicated with arrows. Scale bar = 10 μm.

References

1. Petri WA Jr, Haque R. *Entamoeba* species, including Amebiasis. In: Mandell GL, JE Bennett, R Dolin, eds. Mandell, Douglas, and Bennett's principles and practice of infectious diseases, 7th ed. Philadelphia: Churchill Livingstone; 2010. p. 3411–25.

2. Tamura K, Peterson D, Peterson N, Stecher G, Nei M, Kumar S. MEGA5: molecular evolutionary genetics analysis using maximum likelihood, evolutionary distance, and maximum parsimony methods. Mol Biol Evol. 2011;28:2731–9.

3. Stensvold CR, Lebbad M, Victory EL, Verweij JJ, Tannich E, Alfellani M, et al. Increased sampling reveals novel lineages of Entamoeba: consequences of genetic diversity and host specificity for taxonomy and molecular detection. Protist. 2011;162:525–41.
